# Systematic review reveals multiple sexually antagonistic polymorphisms affecting human disease and complex traits

**DOI:** 10.1111/evo.14394

**Published:** 2021-11-12

**Authors:** Jon Alexander Harper, Tim Janicke, Edward H. Morrow

**Affiliations:** ^1^ Evolution, Behaviour and Environment Group, School of Life Sciences University of Sussex Brighton BN1 9QG United Kingdom; ^2^ Centre d’Écologie Fonctionnelle et Évolutive, UMR 5175, CNRS Université de Montpellier, École Pratique des Hautes Études Montpellier 34293 France; ^3^ Applied Zoology Technical University Dresden Dresden 01062 Germany; ^4^ Department of Environmental and Life Sciences Karlstad University Karlstad SE‐65188 Sweden

**Keywords:** Evolutionary genomics, fitness, selection—sexual, sexual conflict

## Abstract

An evolutionary model for sex differences in disease risk posits that alleles conferring higher risk in one sex may be protective in the other. These sexually antagonistic (SA) alleles are predicted to be maintained at frequencies higher than expected under purifying selection against unconditionally deleterious alleles, but there are apparently no examples in humans. Discipline‐specific terminology, rather than a genuine lack of such alleles, could explain this disparity. We undertook a two‐stage review of evidence for SA polymorphisms in humans using search terms from (i) evolutionary biology and (ii) biomedicine. Although the first stage returned no eligible studies, the second revealed 51 genes with sex‐opposite effects; 22 increased disease risk or severity in one sex but protected the other. Those with net positive effects occurred at higher frequencies. None were referred to as SA. Our review reveals significant communication barriers to fields as a result of discipline‐specific terminology.

In species with separate sexes, an evolutionary conflict at the level of individual genetic loci can occur, where alleles that are favored by selection in one sex are selected against in the other (i.e., intralocus sexual conflict; Parker 1979). This sexually antagonistic (SA) form of selection is thought to be driven by differences in how the two sexes maximize their fitness and concomitant unequal variances in reproductive success. Research into SA selection has expanded recently with the recognition that it feeds into several important evolutionary processes. It is primarily thought to drive the evolution of sexual dimorphism and trait diversification (Lande 1980; Rice 1984; Pennell et al. 2016), including sex‐biased gene expression (Ellegren and Parsch 2007). As a potential driver of balancing selection, it has also been implicated in the maintenance of genetic variation (Connallon and Clark 2013; Grieshop and Arnqvist 2018) that would otherwise be eroded by directional selection. Most recently, it has been suggested that genetic variation at SA loci could contribute to the occurrence of a number of common human diseases (Morrow and Connallon 2013; Morrow 2015), which also show considerable variation between the sexes in terms of their prevalence, severity, and age of onset (Ober et al. 2008; Rigby and Kulathinal 2015).

Despite the interest in sexual antagonism as an evolutionary force, determining the identity of SA loci remains a major challenge (Ruzicka et al. 2020). An early empirical milestone in the field of sexual conflict was achieved by quantitative genetic studies demonstrating that genomes in a laboratory‐adapted population of *Drosophila melanogaster* harbor significant amounts of SA standing genetic variation (Rice 1992; Chippindale et al. 2001). A number of other systems have shown similar results, albeit using different methods, including invertebrates and vertebrates from both lab and wild populations (Foerster et al. 2007; Bonduriansky and Chenoweth 2009; Mills et al. 2012). With the advent of advanced genomic tools, two model systems report specific examples of SA genetic loci: in Atlantic salmon (*Salmo salar*) the VGLL3 locus (Barson et al. 2015), and in *D. melanogaster* the DDT‐R locus (Rostant et al. 2015), the Ala‐278‐Thr polymorphism in the mitochondrial genome (Camus et al. 2015), and multiple candidate loci from a recent genome‐wide association study (Ruzicka et al. 2019).

Although humans have also been shown to experience SA selection for some quantitative traits (Camperio‐Ciani et al. 2004; Garver‐Apgar et al. 2011; Stearns et al. 2012; Stulp et al. 2012), and there are numerous reports of genetic loci with sex‐specific effects (i.e., effects that differ in magnitude between the sexes) on multiple complex traits or disease phenotypes (Gilks et al. 2014; Winkler et al. 2015), there are apparently no clear examples of SA loci in humans, which is inconsistent with theoretical expectations (Connallon and Clark 2014). One potential explanation is that sexual antagonism is a weak selective force in humans, with sexual dimorphism being rather limited (Short 1979; Dixson 2009). This may indicate that concordant selection pressures between the sexes have dominated our evolutionary history. But the recent quantitative genetic studies challenge this view (Stearns et al. 2012; Stulp et al. 2012), and indeed theory predicts there is an inevitability to SA loci occurring in organisms with separate sexes (Parker et al. 1972; Connallon and Clark 2014). Thus, there is a clear disparity between, on the one hand, theoretical expectations and quantitative genetic evidence that SA selection does occur in humans, and on the other hand, a complete lack of specific examples of SA loci. All this in the context of decades of research into how individual genetic variants influence disease profiles.

An alternative explanation for the absence of documented SA loci in humans is that because biomedical science does not use the same terminology for SA effects that evolutionary biology uses, there may in fact be examples that have been misclassified and therefore remain hidden in the literature. For example, sex‐specific effects referred to as sex‐different or sex‐opposite, and sex referred to using gender terms (Khramtsova et al. 2019). Moreover, it is likely that the concepts of (intralocus) sexual conflict and sexual antagonism are not generally well known within biomedical science, and variants that may have SA effects on disease risk may not be referred to as such. A secondary related hypothesis is that SA effects, when discovered, are discounted as errors as they do not match expectations that sex differences in phenotype or genetic architecture are not important (Clayton and Collins 2014). As a result, that may also lead them to be misclassified or simply go unreported, leading to a general publication bias.

For these reasons, we propose that a systematic review of the biomedical literature to identify SA loci in humans requires a specific and targeted set of search terms that would not normally be used within the field of evolutionary biology. We test this assumption by dividing our systematic review into two stages. In the first stage, we search for articles identifying specific genetic loci using terms directly relating to the concept of sexual antagonism and others used in evolutionary biology. We supplement this search with a second stage, where we develop a set of terms that we hypothesize may capture the same concept as sexual antagonism within the biomedical literature, although not explicitly stated as such, as well as additional terminology relating to possible alternatives for describing the two sexes. From our searches, we also extracted data on sex‐specific effect sizes and allele frequencies. We were then able to explore how effect sizes varied for the same alleles between the sexes, and test the prediction that SA alleles experiencing net positive selection between the sexes may achieve higher equilibrium frequencies than alleles that experience more symmetric or net negative patterns of selection (Morrow and Connallon 2013). Our systematic review aims to advance our understanding of SA genes in humans, which show considerable and largely unexplained diversity in sexual dimorphism for disease phenotypes (Ober et al. 2006; Rigby and Kulathinal 2015).

## Methods

For this systematic review, we followed PRISMA guidance where possible (Moher et al. 2009). PubMed (https://pubmed.ncbi.nlm.nih.gov/) was searched for articles on 2 December 2020 with no time limit. The searches were carried out in two stages, with the organism filter set to human in both stages. In Stage 1, eligible studies were required to report specific genetic variants or haplotypes that were referred to as SA or were an example of intralocus sexual conflict. To achieve this, we conducted a Boolean search for articles that used the terms “sexual antagonism” OR “sexually antagonistic” OR “intralocus sexual conflict” AND “locus” OR “loci” OR “gene” OR “snp” OR “polymorphism” OR “variant” OR “allele” in their abstract or title. The Stage 1 search returned 34 articles in total (full search term in the Supporting Information; search output is accessible at https://pubmed.ncbi.nlm.nih.gov/collections/60255050/?sort=pubdate).

**Figure 1:**
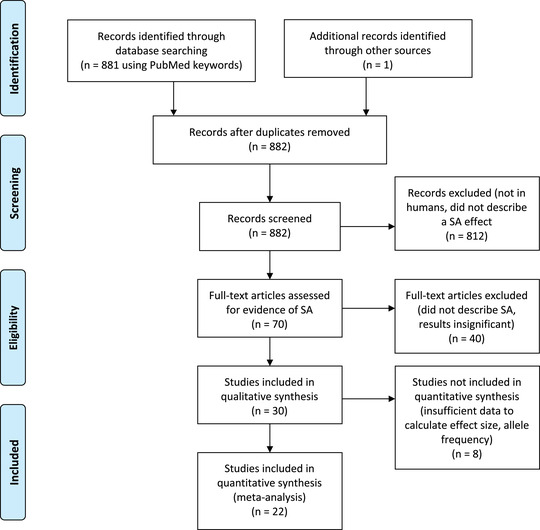
PRISMA flow diagram for systematic review of sexually antagonistic loci in humans.

In Stage 2, studies were required to report specific genetic variants or haplotypes in humans with opposite effects in the two sexes on either complex traits, the outcome of a medical intervention, or disease risk/severity. We define complex traits as likely with a polygenic genetic architecture but are not directly related to a disease phenotype. In this second stage, search terms were specifically designed to include articles from the biomedical literature that may have been missed in the first stage because they do not report their findings with terms normally found within the evolutionary biology literature. Again, we conducted a Boolean search for articles that used terms in their title or abstract to describe an opposite or different effect in the two sexes (“sex dependent,” “sex different,” “gender dependent,” “sex AND opposite,” or “gender AND opposite”), or that capture this concept with alternative words for sex ((“male AND female AND opposite” OR “men AND women AND opposite” OR “boys AND girls AND opposite”) AND (“locus” OR “loci” OR “gene” OR “snp” OR “polymorphism” OR “variant” OR “allele”)). Full details of the search terms used and the numbers of articles returned are provided in the Supporting Information. The Stage 2 search returned 881 articles (Fig. 1) (full search term in the Supporting Information; search output is accessible at https://pubmed.ncbi.nlm.nih.gov/collections/60254985/?sort=pubdate).

The abstracts of the articles from Stage 1 and Stage 2 were then examined, and any articles that had the possibility of reporting an opposite effect of a specific genetic locus on a complex trait, medical intervention, or on disease risk/severity were considered for further screening. From Stage 1, no articles passed the screening. From Stage 2, this screening produced a shortlist of 70 candidate articles (https://pubmed.ncbi.nlm.nih.gov/collections/57906298/?sort=pubdate). Full texts of these candidate articles were then reviewed in detail. Articles were included in the final list if they described a sex‐opposite or SA effect linked to a specific genetic locus or loci, and reported the effect to be statistically significant (at a *P*‐value cutoff of <0.05, or with 95% confidence intervals not overlapping 1). One additional article was considered from an outside source. Studies that only reported significant sex‐by‐variant effects were not automatically included unless they also satisfied the criteria above.

We converted all reported sex‐specific effects into a standard effect size (Cohen's *d*) quantifying the magnitude of how a given variant affects the studied trait expressed in the given sex. Specifically, Cohen's *d* was computed based on the reported descriptive statistics (*N*, mean, standard error) or by conversion from other effect sizes (Odds ratio) and test statistics (*F*‐values, *t*‐values) using formulas reported elsewhere (Borenstein 2009; Gurevitch et al. 2013, pp. 195−206; Lajeunesse 2013). We sought information directly from the authors where these metrics were not possible to extract from the articles themselves (12 authors contacted, five responded, three responded with data, all later excluded as they did not fulfill the criteria for eligibility). We also recorded the gene name, locus (with accession/rs number where possible), trait affected, and the frequency of the focal allele having the effect, hereafter referred to as *effect allele frequency*. Identification for the variants was taken from the studies where possible, but others necessitated searching the National Centre for Biotechnology Information (NCBI) to find the Reference SNP cluster ID for the variants described. Where effect allele frequencies were not reported, we used genotype frequencies to calculate effect allele frequency. Not all studies reported allele or genotype frequencies and so we attempted to supplement these data with allele frequencies from the 1000 genomes database (The 1000 Genomes Project Consortium 2015), because these show a strong correlation with effect allele frequencies reported in the studies reviewed (Pearson correlation: *N* = 25 *r* = 0.94, *P* > 0.001; Fig. S1). However, using this approach we were only able to supplement our allele frequency data for one additional locus (rs7341475 in the *RELN* gene; Table 1). In some cases, loci that affect multiple different traits were found. In such cases, we calculated the geometric mean of their effect sizes to account for possible pseudoreplication.

**Table 1:** Genetic loci in humans showing sexually antagonistic effects on trait expression

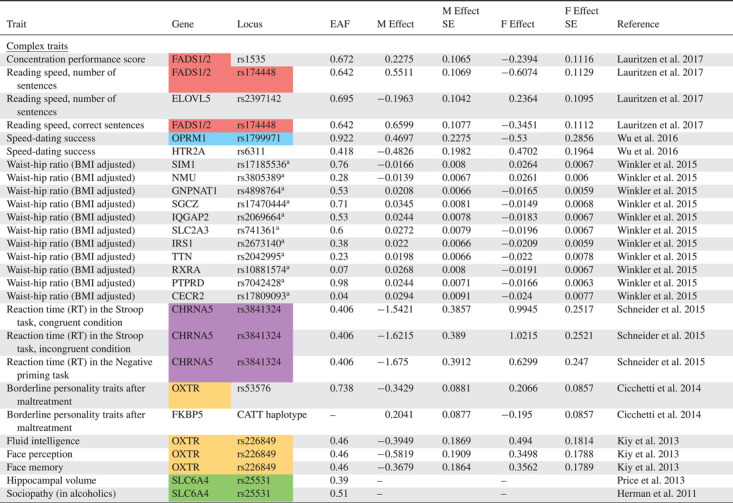
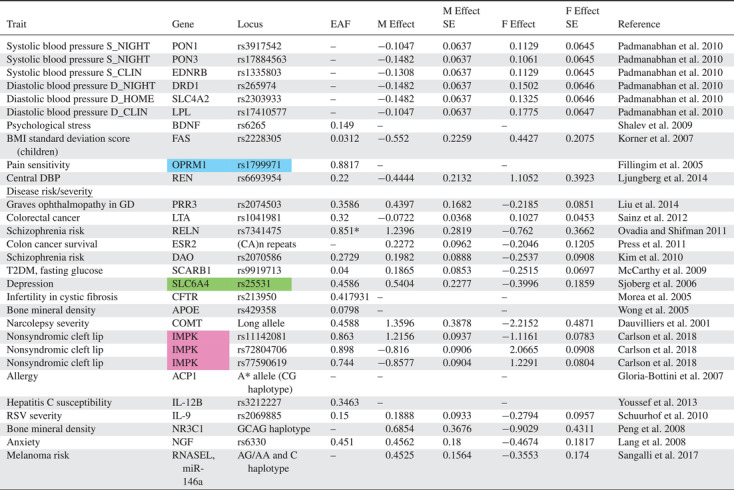

EAF = effect allele frequency (asterisk indicates allele frequency derived from 1000 genomes database); M = male; F = female.

Background colours denote instances where genes and/or variants appear more than once in the list. The superscript “a” denotes alleles from a GWAS (FDR < 5%).

If *b*_f_ and *b*_m_ represent the estimated effect of the focal allele in females and males, respectively, and hence can be represented by the male and female *D* we calculate here, then positive values of *b*_f_ or *b*_m_ imply that the allele is positively associated with disease expression, whereas negative values imply that the allele is negatively associated with disease expression. SA alleles are therefore defined as those with opposite effects between the sexes (i.e., *b*_f_ < 0 < *b*_m_ or *b*_m_ < 0 < *b*_f_). An evolutionary model of evolution at SA loci predicts that as the magnitude of the positive effect that an SA allele has outweighs the negative effect, the higher the frequency that allele can achieve (Morrow and Connallon 2013). We therefore expect a negative relationship between the ratio of positive to negative effect sizes (hereafter referred to as the *effect size ratio*) and observed allele frequency. To investigate this, we calculated the effect size ratio by dividing the positive standardized effect size by the negative standardized effect size; when *b*_f_ < 0 < *b*_m_, effect size ratio was calculated as *b*_m_/*b*_f_, and when *b*_m_ < 0 < *b*_f_, effect size ratio was calculated as *b*_f_/*b*_m_. Thus, SA alleles with a greater beneficial effect will have a smaller, more negative effect size ratio (←1), whereas SA alleles that have a greater deleterious effect will have a larger effect size ratio (>−1), up to a maximum value of 0 (positive values occur when the effect is positive or negative in both sexes, but the allele would then no longer be defined as a SA allele).

We modeled how effect allele frequency changes with effect size ratio using a generalized linear model (GLM), weighted by the inverse of the variance of the effect sizes such that data points with smaller variance have a higher weight, because smaller sample sizes were associated with larger and more variable effect sizes (Fig. S2). We initially also included trait class and its interaction with effect size ratio as a fixed factor with two levels (complex trait and disease trait/severity), because alleles that influence complex traits in opposite directions are not necessarily under SA selection and so may not behave in the way predicted, whereas alleles influencing disease traits, unless very late acting, are more likely to show a closer relationship with marginal effects on fitness. As a response variable, effect allele frequency is limited between 0 and 1, so we looked at allele counts to allow allele frequency to vary freely. GLMs with binomial error distribution showed substantial overdispersion, so we used a quasibinomial function to address this issue (Payne et al. 2018). We used a Chi‐squared test to infer significance of the two predictor variables and their interaction. The full model indicated that the interaction term and trait class have no significant effect, so that we report the fit of the reduced model that only includes effect size ratio. We also subsequently modeled the data for the two trait classes (complex traits and disease risk/severity) separately, again using a quasibinomial distribution function, to see if the result was replicated in these smaller subsets of the data. The raw data and R script are available for replicating the analyses and figures we present (Supporting Information).

## Results

The Stage 1 search found no articles that described genetic loci in humans with effects that were SA. In contrast, the Stage 2 search identified 30 articles that described variants with statistically significant sex‐opposite or SA effects (Fig. 1; https://pubmed.ncbi.nlm.nih.gov/collections/60278165/?sort=pubdate). From the studies examined, 49 SA variants were identified (Table 1), affecting 21 different complex traits (30 loci) and 17 disease risk/severity traits (19 loci), with a large range of effect sizes. There were no variants affecting medical intervention traits that passed the screening process. The majority of alleles had effect sizes of similar absolute values in the two sexes, with larger effects tending to show greater differences on absolute effect size (Fig. S3).

The vast majority of studies we identified in the review (Table 1) investigated associations between traits or disease phenotypes and already known candidate genes, with a single genome‐wide association study (GWAS). An example candidate gene study is Sainz et al. (2012) who investigated the link between colorectal cancer (CRC) and alleles that are known to be associated with type 2 diabetes in an effort to better understand the link between the two diseases. They genotyped 1798 CRC patients and compared these with controls from a population‐based study, focusing on variants previously identified in a GWAS. The study revealed that a SNP in the *LTA* gene was underrepresented in female CRC patients, but more common in males relative to the control population. They reported odds ratios for each sex, which we converted to Cohen's *D*.

A second example of a candidate gene study is Kiy et al. (2013) who focused on two SNPs: rs4680 in *COMT* and rs226849 in the *OXTR* gene. They genotyped 250 participants, who conducted nine different tasks that measured cognitive traits such as face recognition and memory. In three of these tasks, they discovered a significant, opposite effect of the rs226849 SNP on performance in male and female participants. The effect sizes reported were converted from the *R* ^2^ values for each sex.

The single GWAS by Winkler et al. (2015) aimed to identify variants that affect body size and shape, such as body mass index (BMI) and waist‐hip ratio, characterizing how their effects were modulated by age and sex, and included data from 114 studies encompassing up to 320,000 individuals. Forty‐four loci that affect waist‐hip ratio were found to have different effects in men and women, of which 11 had opposite effects in the two sexes. The data reported were beta values stratified by sex, which we converted into Cohen's *D*.

**Figure 2:**
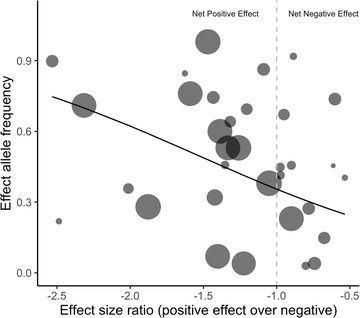
The relationship between effect allele frequency and effect size ratio. Point size varies according to the variance of effect size ratio—larger points have smaller variance and therefore a larger weighting in the model. The vertical dotted line represents the switch point between a net‐negative effect of a particular locus (effect size ratio >−1) and a net positive effect (effect size ratio <−1). The line represents the predicted values derived from the generalized linear model fitted to the data (see *Results*).

We found 22 studies provided sufficient data to allow the relationship between effect size ratio and effect allele frequency to be explored by statistical modeling. Trait class and its interaction with effect size ratio were initially included in the model but neither were found to have a significant effect (GLM: trait class dfs = 1,30, deviance = 15.5 × 10^3^, *P* = 0.968; interaction dfs = 1,29, deviance = 1475, *P* = 0.990) and were therefore excluded from the subsequent model in which effect size ratio was found to relate negatively to effect allele frequency, as predicted (GLM: estimate ± SE = −1.10 ± 0.54, dfs = 1,31, deviance = 38.7 × 10^6^, *P* = 0.036; Fig. 2). For three loci, there were data on effect sizes for more than one trait (Table 1), which introduced a degree of nonindependence between these values in the dataset. However, resampling the data with effect sizes for only one trait for these three loci in turn and modeling these datasets in the same way (18 individual GLMs, dfs = 1,31) did not change the results qualitatively (minimum, maximum: model estimate = −1.0963, −1.0964; deviance = 38.72 × 10^6^, 38.73 × 10^6^; *P* = 0.03618, 0.03620).

Although the interaction term between trait type and effect size ratio was not significant, we wanted to investigate whether the negative relationship between effect size ratio and effect allele frequency across all loci was repeated when the data were divided according to trait class, because although opposite effects on disease risk/severity may show a direct relationship with SA fitness effects, complex traits may or may not be experiencing SA selection, even if effects are in opposite directions in the two sexes. Although the trends were both again negative as predicted, the generalized linear models of these smaller datasets were only marginally nonsignificant (complex traits model estimate ± SE = −1.10 ± 0.69, dfs = 1,19, deviance = 32.6 × 10^6^, *P* = 0.101; disease risk/severity model estimate ± SE = −1.34 ± 0.77, dfs = 1,10, deviance = 5.14 × 10^4^, *P* = 0.053; Fig. S4).

## Discussion

The Stage 1 literature search of our systematic review focused on finding reports of specific genetic loci in humans with evidence for SA effects using terminology normally associated with the evolutionary concepts of sexual conflict or sexual antagonism. The Stage 2 search sought equivalent evidence, but used search terms that we anticipated would be used by scientists outside the field of evolutionary biology, who may not use the same terminology. Although the Stage 1 search did not find any examples of SA loci occurring in humans, the Stage 2 search identified 49 genetic loci across 30 studies that had ostensibly SA effects, but were not described as such. Clearly then the Stage 1 review failed to identify multiple relevant reports going back some 20 years because the terminology used in those reports did not match the conceptual framework of the search. The Stage 2 search may also be a lower limit given searches of other larger databases may also harbor further examples (see below). Although not yet validated, these reports nonetheless represent a substantial body of evidence that humans, like other organisms with separate sexes (Bonduriansky and Chenoweth 2009), inevitably experience SA selection for a wide range of complex traits (Garver‐Apgar et al. 2011; Stearns et al. 2012; Stulp et al. 2012; Connallon and Clark 2014), as well as for a range of diseases.

A key prediction from a population genetic model of SA genetic variation is that SA alleles are expected to achieve higher equilibrium frequencies, without necessarily going to fixation, as the relative magnitude of the positive effect in one sex outweighs the negative effect in the opposite sex, that is, an increasingly negative effect size ratio (Morrow and Connallon 2013). We found clear support for this prediction, with a negative relationship between effect size ratio and allele frequency when examining all complex traits and diseases together (Fig. 2), although this relationship was only marginally significant when complex or disease trait classes were modeled separately (Fig. S3). These results lend support to the view that the loci identified are genuinely experiencing SA selection, and although it is difficult to discern how selection acts on trait size in males and females for complex traits, we would not expect the frequencies of disease‐causing alleles to be so high under a mutation‐selection balance. We were not able to look at the evolutionary history or age of these alleles due to limited information (*n* = 8) (Albers and McVean 2018); it would nonetheless be valuable to examine the population dynamics of these alleles over a broad timescale.

The loci showed a very broad range of effect sizes, from small to very large, with a generally symmetrical inverse relationship between effect size in one sex and the other (Fig. S2), which is generally expected, because our screening process necessarily excluded studies reporting effects in the same direction across the two sexes. Nonetheless, it is striking just how large some of the effects were, with very large negative effects in one sex simultaneously occurring with similarly large positive effects. Larger and more variable effect sizes are expected when sample sizes are small, a pattern we also found, which motivates further investigation of the traits and loci included in this review to elucidate more accurate estimates of real effect sizes.

The loci themselves fall within genes that influence a broad range of phenotypes, including morphological, physiological, and behavioral complex traits, and a similarly diverse range of disease types, including various cancers, neurological disorders, and immune system processes (see Table 1). For the most part, the diseases appear to be early‐acting rather than late‐onset, with the exception of perhaps bone mineral density. As such, it seems reasonable that an allele that increases disease risk or severity in these cases will indeed have a concomitant reduction in marginal fitness. Although a single large study identified several loci related to BMI‐adjusted waist‐to‐hip ratio, there is generally no overall bias toward one particular trait or disease class. The total number of loci is relatively small, however, which may limit the power to identify such biases if they exist. We also did not find any examples of SA loci on the X‐chromosome, which may or may not be a hotspot of sexual antagonism (Rice 1984; Fry 2010; Ruzicka and Connallon 2020). This was expected given that it is commonly not included in genome‐wide analyses (Wise et al. 2013), although most of the articles we reviewed were candidate gene studies. Nonetheless, we anticipate further examples may be identified should data from the X‐chromosome be included systematically in association studies.

We also found evidence that some traits are influenced by more than one genetic variant in sex‐opposite or SA ways (*n* = 8), or that some specific variants have pleiotropic effects on more than one trait or disease in a sex‐opposite or SA way (*n* = 6; see Table 1). This complexity in the genetic architecture of traits or diseases may make conflict resolution particularly difficult, as a change in the allele at a single genetic locus experiencing SA selection may simultaneously influence multiple genes or phenotypes in both sexes in divergent ways (Fitzpatrick 2004; Pennell and Morrow 2013). Consequently, this may explain the persistence of intralocus sexual conflict at these loci. It may be that some of the remaining loci also have as yet unidentified pleiotropic effects with other traits or there are as yet unidentified SA loci influencing those same traits or diseases. Of the 49 variants identified, four were confirmed to be haplotypes. Such variants, consisting of two or more SNPs in linkage disequilibrium having a joint effect, could also present a problem for conflict resolution, because the ability of selection to act on any individual locus independent of the others in the linkage block is reduced for linked loci. This issue may also extend to other single variants reported here if they also occur in linkage blocks.

Although we have identified multiple genetic loci with either sex‐opposite or SA effects, these are likely outnumbered by those with either sex‐specific (same direction but different magnitude) or sex‐limited effects. For instance, Winkler et al. (2015) report 44 loci with sex‐specific or sex‐limited effects but only 11 with sex‐opposite effects. Several other genome‐wide association studies report sex‐specific effects in several human diseases (Khramtsova et al. 2019), and a recent (nonsystematic) review identified 37 SNPs with sex‐dependent effects (Gilks et al. 2014). Detection of loci with SA versus sex‐specific or sex‐limited effects may differ systematically because we expect SA loci are more likely to persist at intermediate frequencies than loci with sex‐specific effects (i.e., that differ between the sexes in magnitude but not sign). A larger systematic review that targeted variants with sex‐specific or sex‐dependent effects would therefore be a valuable contribution and enable us to more clearly understand how important SA alleles are relative to the broader context of genes with nonidentical effects in the two sexes.

A key gap in our knowledge is whether the putative examples of sex‐opposite or SA alleles presented here can be validated using independently derived datasets in the same or different subpopulations. For this, focusing on those variants associated with sex‐differential disease risk would naturally be the most fruitful for advancing our knowledge of sexual antagonism in human disease. We therefore encourage specialists for the particular disease groups presented in Table 1 to include in the future the potential for SA genetic effects to occur when designing studies and analyzing data. It is also important for all studies to report results in sufficient detail so that effect sizes can be calculated (if not given) as well as allele frequency data. Unfortunately, eight studies (representing 17 loci) failed to do this, so could not be included in our review and subsequent test of the evolutionary model. More generally, our study reinforces the view that sex is an important factor in shaping genetic associations with human traits and disease, even in divergent and contradictory ways, and so should always be considered when investigators examine genetic associations with phenotypes (Ober et al. 2008; Lee 2018).

There is a possibility that some bias exists in the PubMed database against articles from the field of evolutionary biology, and if we had used another database then both stages would have returned valid articles. We subsequently (14 December 2020) sought to investigate this possibility with additional searches of Scopus and Web of Science. Repeating the Stage 1 search initially returned 85 articles in Web of Science (Basic search of Topic, refined by “Human” within results) and 71 articles in Scopus (include keywords “Human,” exclude “Non‐Human”), but again no articles made it through the screening process. Repeating the Stage 2 search returned a much larger number of studies in both Web of Science (4573; Basic search of Topic, refined by “Human” within results) and Scopus (6616; Advanced search of Title‐Abstract, filter: include human and humans, exclude nonhumans, Articles only), with 8604 unique records. This large number of records is too many to currently screen and may harbor further examples of SA loci in humans, but it is noteworthy that the majority of the initial list of studies found during Stage 2 using PubMed were also found in the other two databases (712 out of 881) as well as 28 out of the screened list of 32 studies identified in the current systematic review.

The disparity between the two stages of the review process provides a remarkable example of how discipline‐specific terminology and concepts can hinder scientific communication between fields for substantial periods of time. For many of the studies identified in this review, the focus of the research was not on quantifying or even identifying sex‐specific genetic effects in traits or disease, although they were tested for. The responses to such findings varied. Some framed these results as a major finding, whereas others merely made note of them, with one suggesting that because the genetic associations were in opposite directions in the two sexes then it should be regarded as a false positive (Wong et al. 2005). It is possible or even likely then that there exists a publication bias in the biomedical sciences against studies with apparently incongruous sex‐opposite or SA effects.

## CONFLICT OF INTEREST

The authors declare no conflict of interest.

## AUTHOR CONTRIBUTIONS

JAH and EHM conceived the study. JAH performed the searches and created the shortlists. JAH, EHM, and TJ screened the shortlisted articles. JAH and TJ collected and curated data. JAH, EHM, and TJ performed the analysis. JAH and EHM drafted this article, with all authors commenting on revisions.

## DATA ARCHIVING

Stage 1 search output can be found at: https://pubmed.ncbi.nlm.nih.gov/collections/60255050/?sort=pubdate.

Stage 2 search output can be found at: https://pubmed.ncbi.nlm.nih.gov/collections/60254985/?sort=pubdate.

Stage 2 shortlist can be found at: https://pubmed.ncbi.nlm.nih.gov/collections/57906298/?sort=pubdate.

Stage 2 selected articles can be found at: https://pubmed.ncbi.nlm.nih.gov/collections/60278165/?sort=pubdate.

Data and scripts are provided as part of the Supporting Information.

Data and code have been uploaded to Dryad: https://doi.org/10.5061/dryad.rv15dv48k.

Associate Editor: N. G. Prasad

Handling Editor: T. Chapman

## Supporting information


**Figure S1**. Study reported effect allele frequencies and database allele frequencies are strongly correlated.
**Figure S2**. Relationship between study sample size and average extracted effect size across all studies.
**Figure S3**. Female effect size and male effect size are negatively correlated in SA alleles.
**Figure S4**. The relationship between effect allele frequency and effect size ratio, grouped by trait class.
