# Supplementary material for: Systematic review reveals multiple sexually antagonistic polymorphisms affecting human disease and complex traits
Source: Evolution. 2021 Nov 12;75(12):3087–97. doi: 10.1111/evo.14394 (PMC9299215; doi:10.1111/evo.14394)
Supplement: Supplementary file 1 — Figure S1. Study reported effect allele frequencies and database allele frequencies are strongly correlated. Figure S2. Relationship between study sample size and average extracted effect size across all studies. Figure S3. Female effect size and male effect size are negatively correlated in SA alleles. Figure S4. The relationship between effect allele frequency and effect size ratio, grouped by trait class. [file EVO-75-3087-s001.pdf]

Supplementary Material

Both stages of the PubMed literature search employed a Species filter set to Humans only.

Stage 1 Search term:
((((((((((((((((((((sexual antagonism[Title/Abstract] AND locus[Title/Abstract]) OR (sexual antagonism[Title/Abstract] AND loci[Title/Abstract])) OR (sexual antagonism[Title/Abstract] AND gene[Title/Abstract])) OR (sexual antagonism[Title/Abstract] AND snp[Title/Abstract])) OR (sexual antagonism[Title/Abstract] AND polymorphism[Title/Abstract])) OR (sexual antagonism[Title/Abstract] AND variant[Title/Abstract])) OR (sexual antagonism[Title/Abstract] AND allele[Title/Abstract])) OR (sexually antagonistic[Title/Abstract] AND locus[Title/Abstract])) OR (sexually antagonistic[Title/Abstract] AND loci[Title/Abstract])) OR (sexually antagonistic[Title/Abstract] AND gene[Title/Abstract])) OR (sexually antagonistic[Title/Abstract] AND snp[Title/Abstract])) OR (sexually antagonistic[Title/Abstract] AND polymorphism[Title/Abstract])) OR (sexually antagonistic[Title/Abstract] AND variant[Title/Abstract])) OR (sexually antagonistic[Title/Abstract] AND allele[Title/Abstract])) OR (intralocus sexual conflict[Title/Abstract] AND locus[Title/Abstract])) OR (intralocus sexual conflict[Title/Abstract] AND loci[Title/Abstract])) OR (intralocus sexual conflict[Title/Abstract] AND gene[Title/Abstract])) OR (intralocus sexual conflict[Title/Abstract] AND snp[Title/Abstract])) OR (intralocus sexual conflict[Title/Abstract] AND polymorphism[Title/Abstract])) OR (intralocus sexual conflict[Title/Abstract] AND variant[Title/Abstract])) OR (intralocus sexual conflict[Title/Abstract] AND allele[Title/Abstract])

Stage 2 Search term:
(((gender[Title/Abstract] AND opposite[Title/Abstract] AND locus[Title/Abstract]) OR (gender[Title/Abstract] AND opposite[Title/Abstract] AND loci[Title/Abstract])) OR (gender[Title/Abstract] AND opposite[Title/Abstract] AND gene[Title/Abstract])) OR (gender[Title/Abstract] AND opposite[Title/Abstract] AND snp[Title/Abstract])) OR (gender[Title/Abstract] AND opposite[Title/Abstract] AND polymorphism[Title/Abstract])) OR (gender[Title/Abstract] AND opposite[Title/Abstract] AND variant[Title/Abstract])) OR (sex[Title/Abstract] AND opposite[Title/Abstract] AND locus[Title/Abstract])) OR (sex[Title/Abstract] AND opposite[Title/Abstract] AND loci[Title/Abstract])) OR (sex[Title/Abstract] AND opposite[Title/Abstract] AND gene[Title/Abstract])) OR (sex[Title/Abstract] AND opposite[Title/Abstract] AND snp[Title/Abstract])) OR (sex[Title/Abstract] AND opposite[Title/Abstract] AND polymorphism[Title/Abstract])) OR (sex[Title/Abstract] AND opposite[Title/Abstract] AND variant[Title/Abstract])) OR (sex dependent[Title/Abstract] AND locus[Title/Abstract])) OR (sex dependent[Title/Abstract] AND loci[Title/Abstract])) OR (sex dependent[Title/Abstract] AND gene[Title/Abstract])) OR (sex dependent[Title/Abstract] AND snp[Title/Abstract])) OR (sex dependent[Title/Abstract] AND polymorphism[Title/Abstract])) OR (sex dependent[Title/Abstract] AND

variant[Title/Abstract])) OR (sex different[Title/Abstract] AND locus[Title/Abstract])) OR (sex different[Title/Abstract] AND loci[Title/Abstract])) OR (sex different[Title/Abstract] AND gene[Title/Abstract])) OR (sex different[Title/Abstract] AND snp[Title/Abstract])) OR (sex different[Title/Abstract] AND polymorphism[Title/Abstract])) OR (sex different[Title/Abstract] AND variant[Title/Abstract])) OR (gender-dependent[Title/Abstract] AND locus[Title/Abstract])) OR (gender-dependent[Title/Abstract] AND loci[Title/Abstract])) OR (gender-dependent[Title/Abstract] AND gene[Title/Abstract])) OR (gender-dependent[Title/Abstract] AND snp[Title/Abstract])) OR (gender-dependent[Title/Abstract] AND polymorphism[Title/Abstract])) OR (gender-dependent[Title/Abstract] AND variant[Title/Abstract])) OR (male[Title/Abstract] AND female[Title/Abstract] AND opposite[Title/Abstract] AND locus[Title/Abstract])) OR (male[Title/Abstract] AND female[Title/Abstract] AND opposite[Title/Abstract] AND loci[Title/Abstract])) OR (male[Title/Abstract] AND female[Title/Abstract] AND opposite[Title/Abstract] AND gene[Title/Abstract])) OR (male[Title/Abstract] AND female[Title/Abstract] AND opposite[Title/Abstract] AND snp[Title/Abstract])) OR (male[Title/Abstract] AND female[Title/Abstract] AND opposite[Title/Abstract] AND polymorphism[Title/Abstract])) OR (male[Title/Abstract] AND female[Title/Abstract] AND opposite[Title/Abstract] AND variant[Title/Abstract])) OR (men[Title/Abstract] AND women[Title/Abstract] AND opposite[Title/Abstract] AND locus[Title/Abstract])) OR (men[Title/Abstract] AND women[Title/Abstract] AND opposite[Title/Abstract] AND loci[Title/Abstract])) OR (men[Title/Abstract] AND women[Title/Abstract] AND opposite[Title/Abstract] AND gene[Title/Abstract])) OR (men[Title/Abstract] AND women[Title/Abstract] AND opposite[Title/Abstract] AND snp[Title/Abstract])) OR (men[Title/Abstract] AND women[Title/Abstract] AND opposite[Title/Abstract] AND polymorphism[Title/Abstract])) OR (men[Title/Abstract] AND women[Title/Abstract] AND opposite[Title/Abstract] AND variant[Title/Abstract])) OR (boys[Title/Abstract] AND girls[Title/Abstract] AND opposite[Title/Abstract] AND locus[Title/Abstract])) OR (boys[Title/Abstract] AND girls[Title/Abstract] AND opposite[Title/Abstract] AND loci[Title/Abstract])) OR (boys[Title/Abstract] AND girls[Title/Abstract] AND opposite[Title/Abstract] AND gene[Title/Abstract])) OR (boys[Title/Abstract] AND girls[Title/Abstract] AND opposite[Title/Abstract] AND snp[Title/Abstract])) OR (boys[Title/Abstract] AND girls[Title/Abstract] AND opposite[Title/Abstract] AND polymorphism[Title/Abstract])) OR (boys[Title/Abstract] AND girls[Title/Abstract] AND opposite[Title/Abstract] AND variant[Title/Abstract])) OR (gender[Title/Abstract] AND opposite[Title/Abstract] AND allele[Title/Abstract])) OR (sex[Title/Abstract] AND opposite[Title/Abstract] AND allele[Title/Abstract])) OR (sex dependent[Title/Abstract] AND allele[Title/Abstract])) OR (sex different[Title/Abstract] AND allele[Title/Abstract])) OR (gender-dependent[Title/Abstract] AND allele[Title/Abstract])) OR (male[Title/Abstract] AND female[Title/Abstract] AND opposite[Title/Abstract] AND allele[Title/Abstract])) OR (men[Title/Abstract] AND women[Title/Abstract] AND opposite[Title/Abstract] AND allele[Title/Abstract])) OR (boys[Title/Abstract] AND girls[Title/Abstract] AND opposite[Title/Abstract] AND allele[Title/Abstract])

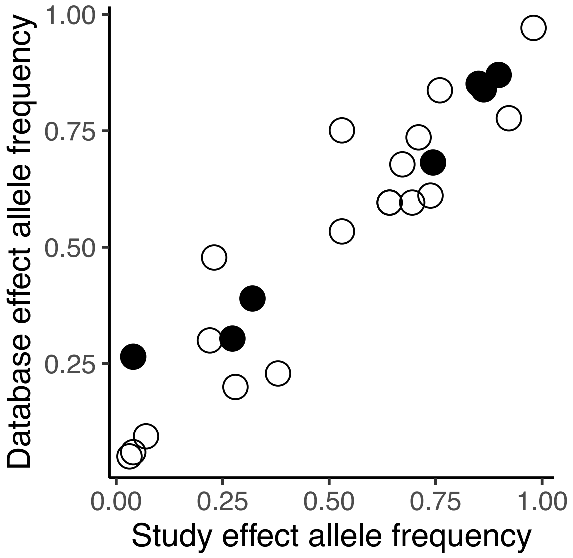

Figure S1. Study reported effect allele frequencies and database allele frequencies are strongly correlated. The correlation between reported effect allele frequency and frequencies for the same alleles obtained from the 1000 genomes database (0.94). Filled circles represent disease risk/severity related variants while open circles represent complex trait variants.

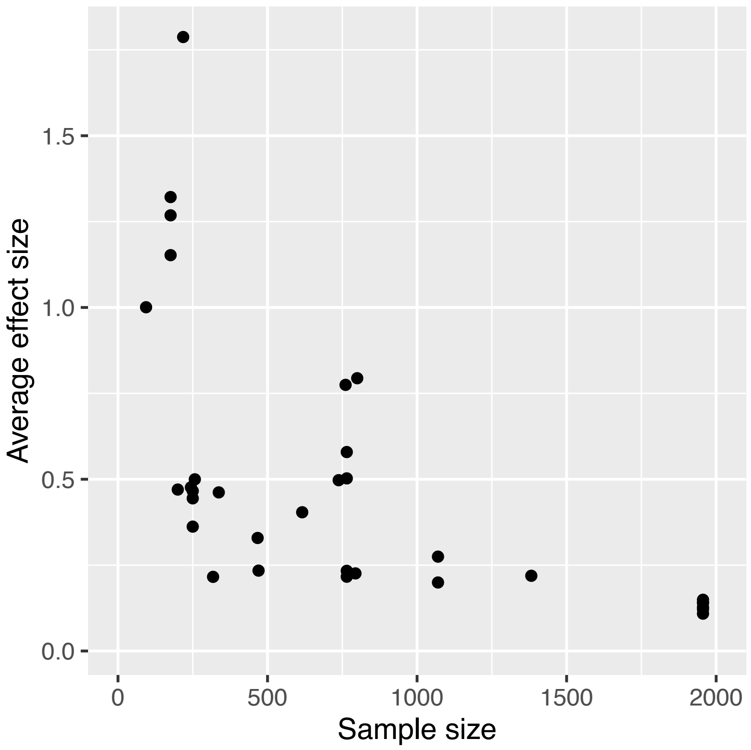

Figure S2. Relationship between study sample size and average extracted effect size across all studies.

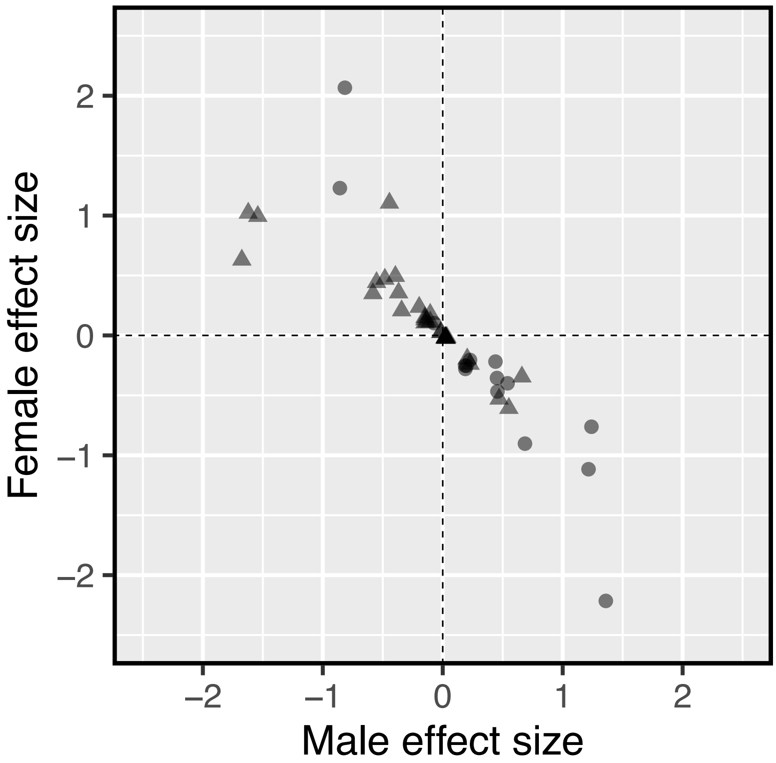

Figure S3. Female effect size and male effect size are negatively correlated in SA alleles. Effect size (Cohen's D) of variants in females against effect size in males. Triangular points represent sex-opposite complex trait variants, circular points represent disease risk/severity variants.

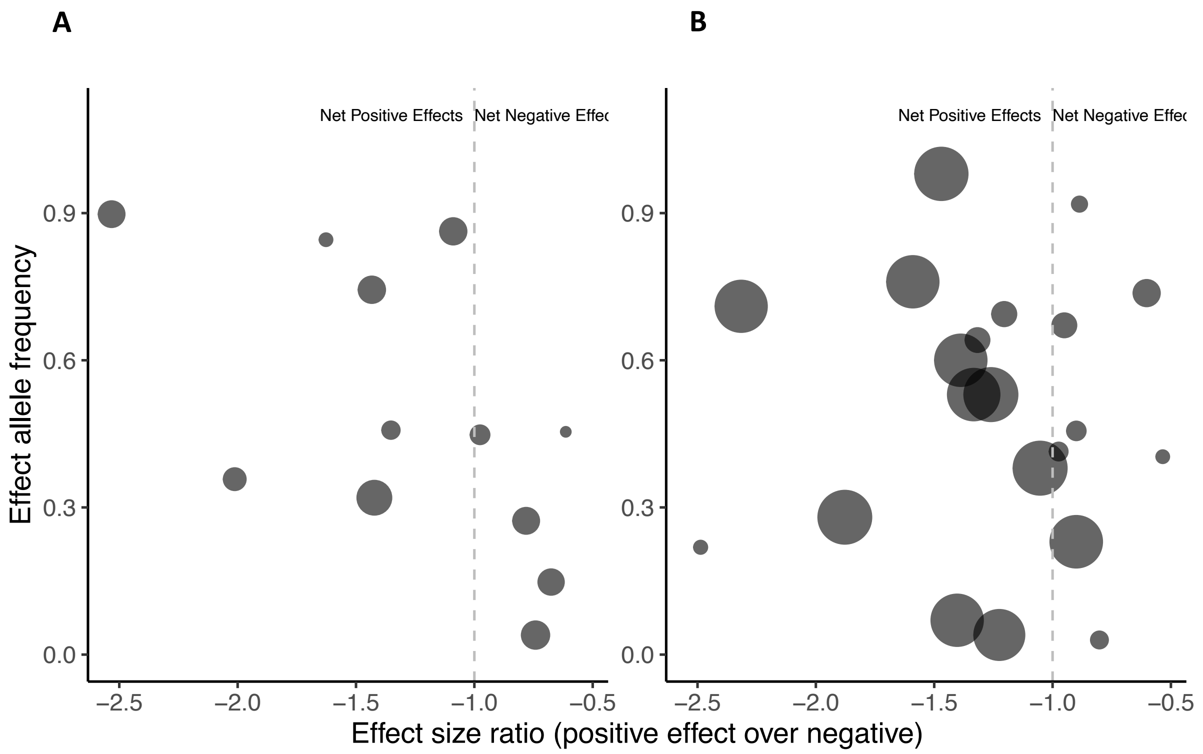

Figure S4. The relationship between effect allele frequency and effect size ratio, grouped by trait class. Points to the right of the vertical dotted line have a greater negative effect than positive. **A.** Disease risk/severity variants. **B.** Complex traits. Point size is based on the variance of the effect size ratio, with smaller variance having larger point sizes and greater weighting in the statistical model.
